# Comparison of Four fMRI Paradigms Probing Emotion Processing

**DOI:** 10.3390/brainsci11050525

**Published:** 2021-04-21

**Authors:** Corinna Hartling, Sophie Metz, Corinna Pehrs, Milan Scheidegger, Rebecca Gruzman, Christian Keicher, Andreas Wunder, Anne Weigand, Simone Grimm

**Affiliations:** 1 Department of Psychiatry and Psychotherapy, CBF, Charité Universitätsmedizin Berlin, 12203 Berlin, Germany; sophie.metz@charite.de (S.M.); rebecca.gruzman@fu-berlin.de (R.G.); simone.grimm@charite.de (S.G.); 2 Bernstein Center for Computational Neuroscience, Humboldt-University Berlin, 10115 Berlin, Germany; corinna.pehrs@bccn-berlin.de; 3 Department of Psychiatry, Psychotherapy and Psychosomatics, University of Zurich, 8032 Zurich, Switzerland; milan.scheidegger@bli.uzh.ch; 4 Charité Research Organisation GmbH, 10117 Berlin, Germany; Christian.keicher@charite-research.org; 5 Translational Medicine and Clinical Pharmacology, Boehringer Ingelheim Pharma GmbH and Co. KG, 52216 Ingelheim am Rhein, Germany; andreas.wunder@boehringer-ingelheim.com; 6 Department of Psychology, Medical School Berlin, 14197 Berlin, Germany; anne.weigand@hu-berlin.de

**Keywords:** fMRI paradigms, emotion processing, amygdala, anterior insula, pregenual ACC

## Abstract

Previous fMRI research has applied a variety of tasks to examine brain activity underlying emotion processing. While task characteristics are known to have a substantial influence on the elicited activations, direct comparisons of tasks that could guide study planning are scarce. We aimed to provide a comparison of four common emotion processing tasks based on the same analysis pipeline to suggest tasks best suited for the study of certain target brain regions. We studied an n-back task using emotional words (EMOBACK) as well as passive viewing tasks of emotional faces (FACES) and emotional scenes (OASIS and IAPS). We compared the activation patterns elicited by these tasks in four regions of interest (the amygdala, anterior insula, dorsolateral prefrontal cortex (dlPFC) and pregenual anterior cingulate cortex (pgACC)) in three samples of healthy adults (N = 45). The EMOBACK task elicited activation in the right dlPFC and bilateral anterior insula and deactivation in the pgACC while the FACES task recruited the bilateral amygdala. The IAPS and OASIS tasks showed similar activation patterns recruiting the bilateral amygdala and anterior insula. We conclude that these tasks can be used to study different regions involved in emotion processing and that the information provided is valuable for future research and the development of fMRI biomarkers.

## 1. Introduction

A variety of different fMRI paradigms have been used to probe emotion processing in order to understand its neural underpinnings in healthy subjects [1] and to study the effects that development [2], psychopathology [3] or therapeutic interventions [4,5] exert on it. The differences between these paradigms lie in the stimulus material used (e.g., printed words, pictures, melodies), the task posed to participants (e.g., passive viewing, matching, emotional Stroop or n-back) and the cognitive effort needed to fulfill the task (e.g., 0-back versus 2-back conditions, emotional judgements). Previous research has shown that the nature of the task and stimuli employed have a considerable impact on the effects [6,7,8] and that different tasks can trigger different aspects of emotion processing [9]. In a few instances, even tasks intended to represent the same concept appear to elicit different activation patterns: a study comparing amygdala activation in four different threat reactivity tasks found that amygdala activation did not correlate significantly across the tasks [10]. Importantly, standard emotion processing paradigms that aim to study certain target regions or aspects have not been established yet and direct comparisons between tasks are scarce. This can pose a challenge for planning new experiments. Furthermore, fMRI studies often differ in their pre-processing routines and analysis software so that activation differences cannot safely be ascribed solely to the task at use. The goal of the present study was to compare common emotion processing fMRI tasks based on the same analysis pipeline regarding the activation they elicited in core regions of emotion processing. 

Meta-analyses of fMRI research on emotion processing have robustly implicated several brain regions, namely, the amygdala, the anterior insula, the pregenual and subgenual anterior cingulate cortices (ACC) as well as the dorsal ACC (dACC), the dorsomedial prefrontal cortex (dmPFC), the dorsolateral PFC (dlPFC), the parahippocampus, the orbitofrontal cortex and visual and auditory cortices [1,9,11]. The constructionist approach [11] assumes that emotion processing draws on more basic psychological operations and therefore recruits the respective brain networks underlying these operations. By this account, the brain regions activated in fMRI tasks of emotion can each be associated with a functional network that exerts a subprocess of emotion. The functional networks assumed to work together in emotion processing are the limbic network (realizing affective states in the body), salience network (detecting behaviorally relevant information), default-mode network (self-referential conceptualization of information) and the executive control network (evaluating or manipulating the incoming information [11]). 

A recent study on a databank of task-based fMRI studies of emotion processing clustered studies based on similar activation patterns elicited by the task in use then performed meta-analyses for each of the clusters of studies. This approach dissociated five brain networks with convergent activations during different types of emotion processing tasks [9]. Apart from two networks in the sensory cortices, these were largely overlapping with the salience, default mode and limbic networks. Subsequently, the meta-data of the experimental designs in each cluster were analyzed. Based on this, the found networks were characterized as contributing to drawing attention to salient information, appraisal and prediction of emotional information and induction of the emotional response, respectively. These results are in line with the constructionist view [11] that emotion processing draws on psychological functions engendered by large-scale brain networks. 

Therefore, as regions of interest we chose one hub of each of these networks: the amygdalae, anterior insulae, pregenual ACC (pgACC) and bilateral dlPFC. The amygdala is the region that is most robustly engaged in emotion processing [1,6,7] and shows the greatest functional connectivity with other regions involved in emotion processing [1]. It is involved in signaling whether sensory information is motivationally salient [12] and has also been thought of as realizing a “core affect”, i.e., affective bodily sensations [11]. In clinical neuroscience, an altered amygdala function in emotion processing, specifically hyperactivation to negative stimuli, has been found in patients with depression, social anxiety, post-traumatic stress and borderline personality disorder [3,13,14].

The anterior insula is essential for the awareness of interoceptive information [15] as well as affective experience [16] and robustly activates during the perception of emotional stimuli [17]. It is a core component of the salience network [18] where it proposedly integrates physiological information with emotional, cognitive and motivational signals to detect the salience of stimuli. Therefore, the activation of the anterior insula in emotion processing may be linked to the salience of emotional stimuli. The pregenual ACC (pgACC) is a part of the default mode network and is typically deactivated in goal-directed tasks [19] but is activated during self-referential thought [20] and the assessment of internal emotional states [21] as well as emotion perception [22]. It has been suggested to subserve a hub function integrating emotion and cognition through its projections to several cortical regions [23] and its involvement in emotion processing may reflect a cognitive appraisal of the emotional content of stimuli [9,24]. The pregenual ACC seems to play a crucial role in the cognitive regulation of emotions [20]. The dlPFC is a core region in the fronto-parietal control network that supports executive attention, working memory and complex problem-solving [18]. It has been found to activate in emotion processing tasks especially when participants are asked to categorize or evaluate emotional information [11]. DlPFC activity competes with amygdala activity in tasks that present an interference of emotional content and cognitive demand [25] such that cognitive load is negatively correlated with amygdala activation in the presence of emotional stimuli [26]. Consistent with these findings, dlPFC activity has been associated with emotion regulation [27].

In this study we aimed to assess the different brain activation profiles elicited by four different tasks that have been widely used within the affective neurosciences. Thereby, we hope to inform the choice of experimental designs when aiming to examine specific parts of the emotion processing network. One task was an emotional working memory paradigm (EMOBACK [28]) and three were passive emotion viewing tasks with attentional control. One of these used emotional face stimuli (FACES task) and the two others used pictures of emotional scenes that were either positive and negative (IAPS) or solely negative (OASIS). All tasks used stimulus material from validated sets of pictures or words whose emotional valence has been established. We analyzed three datasets to investigate how the selected four core regions activated in response to the specific kind of emotional stimulation in each of the tasks.

## 2. Materials and Methods

### 2.1. Participants 

This study analyses data from 45 healthy males aged 18–58 belonging to three samples each consisting of 15 subjects. The mean age of the participants was 25.8 (±5.3) years for the sample from whom FACES and OASIS task data were collected, 29.3 (±2.9) years for the EMOBACK task sample and 35.5 (±10.8) years for the IAPS task sample. Supplementary Table S1 shows the data on the demographic variables and scanning sites. Exclusion criteria were standard MR exclusion criteria, cardiovascular diseases, recent heart or head surgery, current pregnancy, history of psychiatric or neurological disorders and current use of any psychoactive medication. The study was conducted according to the latest version of the Declaration of Helsinki. The full procedure and purpose were explained to each subject in detail as approved by the institutional review boards before they gave written informed consent to enter the study. 

### 2.2. Tasks

#### 2.2.1. EMOBACK Task 

The EMOBACK task [28] is an emotional 2-back task that uses verbal stimuli selected from the Berlin Affective Word List (BAWL [29]). Subjects were required to monitor a series of words and to respond every time a word was presented that was identical to the one presented two trials previously. They were instructed to respond as quickly and as accurately as possible. The stimuli were categorized as either positive, negative or neutral and were matched with regard to length, imageability, emotional arousal and frequency of appearance. The stimuli were presented in 15 blocks; five for each valence category (positive, negative or neutral). Between the block a fixation cross appeared for 10–14 s. Each block contained 15 words presented for 500 ms each. The interstimulus interval was 1500 ms long. A brief training of the task outside the scanner preceded the scanning session. The task lasted for 12 min.

#### 2.2.2. FACES Task

In the FACES task, participants were shown pictures from the Warsaw Set of Emotional Facial Expression Pictures (WSEFEP [30]). The block design task consisted of twelve blocks with six negative emotional faces displaying sadness, fear and disgust (in randomized order) and twelve blocks with scrambled faces (control condition). In total, 72 negative faces of 24 actors (50% female) were shown for 3 s each. The inter-trial interval was jittered between 10 ± 1 s. During the inter-trial interval (ITI), participants viewed a white fixation cross on a black background. To ensure attention, participants were asked to indicate by a button press whether the person was female for the portraits or, for the scrambled faces, whether the colored frame around the picture was blue (compared with green). The paradigm lasted 13 min. A brief training of the task outside the scanner preceded the scanning session.

#### 2.2.3. IAPS Task 

The IAPS task consisted of 80 photographs (40 positive and 40 negative) from the International Affective Picture System [31] presented in a block design. Five pictures were shown during a block of 20 s duration. To ensure attention, participants were presented with a question after each block regarding the content of one of the five pictures for 8 s (e.g., ‘Was there a cat in the picture?’). After the rating, a fixation cross was shown for 20 s to serve as a baseline condition. The fMRI paradigm was composed of 16 blocks (eight positive and eight negative) with an overall duration of 13 min. 

#### 2.2.4. OASIS Task

The OASIS task is a passive picture viewing task with attentional control. During the task, scenes from the OASIS picture set [32] with a negative valence and high arousal rating were presented in a block design. Scrambled pictures were used in a neutral control condition. There were 14 negative and 14 neutral blocks, each lasting 18 s. Within each block, three picture stimuli were presented consecutively for 6 **s** each, resulting in a set of 42 negative pictures and 42 scrambled pictures in total. The order of the blocks was semi-randomized. The inter-trial interval was jittered within a range of 10 ± 1 s to ensure the reduced predictability of picture onset and optimized sampling of the BOLD signal [33]. During the ITI, participants saw a fixation cross. To ensure attention, participants were asked to indicate by a button press whether there was a person present in the picture or for the scrambled pictures whether the bounding box was blue or green. A brief training of the task outside the scanner preceded the scanning session. The experiment lasted 15 min. 

Supplementary Table S2 gives an overview of all task characteristics for the four tasks and Supplementary Figure S1 shows example stimuli from each task. All tasks were presented via MRI compatible video goggles (VisuaStim digital, Resonance Technology, Inc., Los Angeles, CA, USA) using Presentation^®^ (Neurobehavioral Systems, Inc., Albany, CA, USA). Participants responded by pushing a fiber-optic light sensitive key press.

### 2.3. Data Collection

Imaging was performed using 3T MR systems at three study sites (Berlin Center for Advanced Neuroimaging (BCAN), Center for Cognitive Neuroscience Berlin (CCNB) and University of Zurich (UZH). The exact scanner type and sequence parameters at each site can be found in Supplementary Table S3. For each sample, scanning consisted of functional imaging by a T2-weighted echo planar imaging sequence and one anatomical reference image using a 3-dimensional T1-weighted scan. The FACES and OASIS tasks were assessed in one session following a 3D scan. For the two other tasks, subjects completed a 3D scan and one task-based functional scan (EMOBACK and IAPS, respectively). Imaging for all four tasks was collected in one run. 

### 2.4. Data Analysis 

For behavioral data, accuracy was defined as accuracy = #correct responses/#trials for the FACES, OASIS and IAPS tasks and as accuracy = (#hits-#false alarms)/#targets in the EMOBACK task. A threshold of 80% accuracy for the FACES, OASIS and IAPS tasks and 50% accuracy for the EMOBACK task was defined for participants to be included in the data analysis. 

FMRI data were analyzed using MATLAB 2020a (The Mathworks Inc., Natick, MA, USA) and SPM12 revision 7771 (Statistical parametric mapping software, SPM; Wellcome Department of Imaging Neuroscience, London, UK; http://www.fil.ion.ucl.ac.uk (accessed on 4 January 2020)). The first five volumes of each run were discarded to allow for T1 stabilization. The following pre-processing steps were realized: realignment according to the first volume for motion correction, normalization to a standard stereotactic space template from the Montreal Neurological Institute (MNI) and spatial smoothing using a 6 mm FWHM Gaussian kernel. The time series were high-pass filtered (filter width 128 s) to eliminate low-frequency components and adjusted for systematic differences across the trials. We checked for artifacts and set a cut-off for motion parameters at 3 mm or 3°; all volumes of all subjects passed this check. A statistical analysis on the subject level was performed by modeling the different conditions convolved with a hemodynamic response function as explanatory variables within the context of the general linear model on a voxel-by-voxel basis [34]. Realignment parameters were included as additional regressors in the statistical model. A fixed-effect model was performed to create images of parameter estimates, which were then entered into a second-level random-effects analysis. For the fMRI data group analysis, the contrast images from the analysis of the individual participants were analyzed using one-sample *t*-tests.

For each subject, a contrasts testing response to emotional stimuli relative to the baseline or neutral stimuli was calculated. Specifically, for the four tasks these were: 1. EMOBACK: emotional stimuli versus the fixation condition (Emotional > break); 2. OASIS: emotional stimuli versus the control condition (Emotional > scrambled); 3. IAPS: emotional stimuli versus the fixation condition (Emotional > break); 4. FACES: emotional stimuli versus the control condition (Emotional > scrambled).

**Figure 1:**
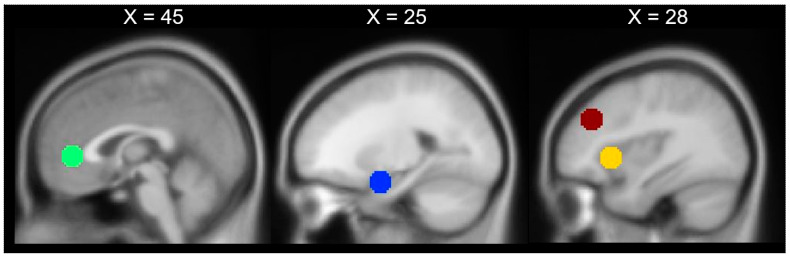
Region of interest (ROI) templates were spheres with a 10 mm diameter. Red = dorsolateral prefrontal cortex, yellow = anterior insula, green = pregenual anterior cingulate cortex, blue = amygdala.

Regions of interest (ROIs) were defined to examine emotion-related brain activations. Specifically, the following ROIs previously linked to emotion processing [1,11] were selected (abbreviation and MNI coordinates in brackets): the bilateral dorsolateral prefrontal cortex (l/rdlPFC; ± 40 36 32), the bilateral amygdala (l/rAM ± 24-2-20), the bilateral anterior insula (l/rAI ± 34 20 0) and the pregenual anterior cingulate cortex (pgACC; 0 42 2). Spherical ROI templates with a diameter of 10 mm were built with automated term-based meta-analyses on neurosynth.org or based on our own previous studies (for pgACC [28]). All ROIs are illustrated in Figure 1. The mean parameter estimate of each ROI was extracted using the REX Toolbox (https://www.nitrc.org/projects/rex/, accessed on 4 May 2020). Significance tests for the ROI analyses were conducted with an α-level Bonferroni adjusted for the number of ROI of *p* < 0.05/7 = *p* < 0.0071.

Paired and independent sample *t*-tests were conducted to compare activation in the ROI between the tasks that had been collected in the same and different samples, respectively. As exploratory analyses, these were reported at an uncorrected α-level of *p* = 0.05. 

## 3. Results

**Figure 2:**
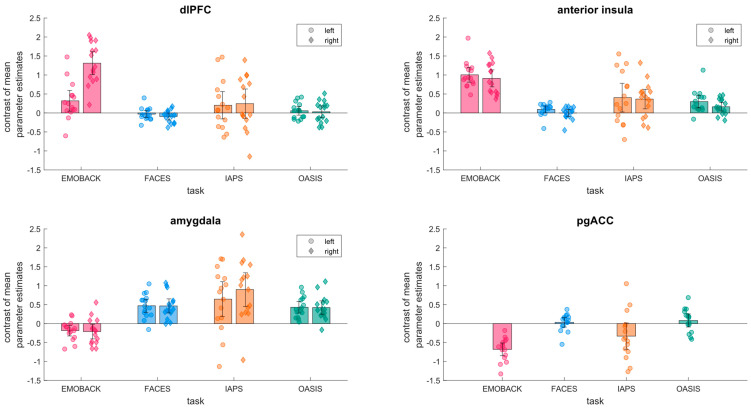
Mean parameter estimates of neuronal activation in the prespecified regions of interest on the group (bars) and individual (dots) level. Error bars represent the 95% confidence interval. Colors represent the four tasks; pink: EMOBACK (Emotional > break), blue: FACES (Negative > scrambled), orange: IAPS (Emotional > break), green: OASIS (Negative > scrambled).

Figure 2 shows an overview of the activations in our regions of interest for all four tasks. Activations in response to the emotional working memory condition compared with the break in the EMOBACK task were found in the bilateral anterior insula and right dorsolateral prefrontal cortex along with a significant decrease in activity in the pgACC (pgACC: mean *β* = −0.681, 95% CI (−0.512, −0.849), t(14) = −8.666, *p* < 0.00001; lAI: mean *β* = 1.004, 95% CI (0.810, 1.199), t(14) = 11.110, *p* < 0.00001; rAI mean *β* = 0.910, 95% CI (0.686, 1.135), t(14) = 8.713, *p* < 0.00001; rdlPFC: mean *β* = 1.310, 95% CI (1.010, 1.611), t(14) = 9.341, *p* < 0.00001). There was also a noticeable activation in the left dlPFC that was, however, not significant to a Bonferroni corrected alpha-level (ldlPFC: mean *β* = 0.320, 95% CI (0.046, 0.593), t(14) = 2.505, *p* = 0.0252).

The FACES task elicited a significant activation in the bilateral amygdala (lAM: mean *β* = 0.469, 95% CI = (0.296, 0.644), t(14) = 5.783, *p* = 0.00005; rAM: mean *β* = 0.466, 95% CI (0.278, 0.652), t(14) = 5.365, *p* = 0.00001). 

The IAPS task elicited an activation in the bilateral amygdala and bilateral anterior insula during the presentation of emotional stimuli (the activation in the left amygdala and bilateral anterior insula were not significant to a Bonferroni adjusted alpha-level of *p* = 0.007; rAM: mean *β* = 0.898, 95% CI (0.454, 0.631), t(14) = 4.33, *p* = 0.0007; lAM: mean *β* = 0.646, 95% CI (0.186, 1.106), t(14) = 3.015, *p* = 0.009; lAI: mean *β* = 0.404, 95% CI = (0.026, 0.781), t(14) = 2.295, *p* = 0.038; rAI: mean *β* = 0.366, 95% CI = (0.108, 0.624), t(14) = 3.043, *p* = 0.009). 

The activation elicited by emotional stimuli in the OASIS task was found in the bilateral amygdala as well as in the bilateral anterior insula (Bonferroni corrected significance not met for rAI; lAM: mean *β* = 0.431, 95% CI (0.278, 0.584), t(14) = 6.063, *p* = 0.00003; rAM: mean *β* = 0.422, 95% CI (0.245, 0.601), t(14) = 5.103, *p* = 0.00016; lAI: mean *β* = 0.299, 95% CI = (0.133, 0.465), t(14) = 3.882, *p* = 0.002; rAI: mean *β* = 0.163, 95% CI (0.050, 0.276), t(14) = 3.091, *p* = 0.008).

As data for the FACES and the OASIS task stemmed from the same subjects, we further conducted exploratory paired *t*-tests to directly compare the activation patterns between the two tasks. No significant difference arose except for the right anterior insula where the activation in the OASIS task exceeded that in the FACES task (OASIS: M = 0.163 ± 0.041; FACES: M = 0.002 ± 0.027; Cohen’s d = 0.633; *p* = 0.028). 

Furthermore, we also conducted exploratory independent *t*-tests comparing the activation elicited in each of the regions of interest between the tasks that were run in different samples. In the right dlPFC, the activation elicited by EMOBACK was found to be significantly stronger than that under any other task (EMOBACK: M = 1.310 ± 0.525; IAPS M = 0.245 ± 0.673; FACES: M = 0.094 ± 0.159; OASIS: M = 0.031 ± 0.270; Cohen’s d >1.7; all *p* < 0.001 for all comparisons of EMOBACK with other tasks). In the left dlPFC, the only significant difference that arose was between EMOBACK (M = 0.320 ± 0.477) and the FACES task (M: 0.030 ± 0.162; Cohen’s d = 0.982; *p* = 0.015). 

In both the left and right amygdalae, EMOBACK elicited significantly less activation than the other tasks (EMOBACK lAM: M = −0.185 ± 0.255 rAM = −0.210 ± 0.347 Cohen’s d > 1.4, *p* < 0.001 for all comparisons with other tasks); in the right amygdala, the activation elicited by IAPS was also significantly stronger than that in the OASIS task. (IAPS: M = 0.898 ± 0.775 OASIS M = 0.422 ± 0.310 Cohen’s d = 0.805, *p* = 0.042) 

In the anterior insula, the activation elicited by EMOBACK was found to be significantly greater bilaterally than that by any other task (EMOBACK rAI: M = 0.910 ± 0.404 lAI: M = 1.004 ± 0.350 Cohen’s d > 1.1, *p* < 0.006 for all comparisons). For the right anterior insula, a significant difference was found also between the IAPS and the FACES task, (IAPS: M = 0.365 ± 466 FACES: M = −0.003 ± 0.158 Cohen’s d = 1.094, *p* = 0.007) with greater activation elicited by the IAPS task. 

In the pgACC, no significant difference was found between the deactivations elicited by EMOBACK or the IAPS task; however, both differed significantly from the other two tasks, FACES and OASIS (EMOBACK M: −0.681 ± 0.294 IAPS: M = −0.331 ± 0.606 FACES: M = 0.033 ± 0.219 OASIS: M = 0.082 ± 0.301 Cohen’s d > 0.75, *p* < 0.05 for all comparisons of EMOBACK or the IAPS task with the OASIS and FACES tasks).

## 4. Discussion

We compared four fMRI paradigms that are often applied in the affective neurosciences regarding their potential of eliciting an activation in regions commonly associated with emotion processing. Our results indicated that the different fMRI paradigms elicited different neural activation patterns. The EMOBACK task elicited activation in the right dlPFC and the bilateral anterior insula and deactivation in the pgACC (but no significant change in amygdala activation). The FACES task induced activity selectively in the bilateral amygdala and the two tasks that used emotionally valenced scenes, OASIS and IAPS, both induced activity in the bilateral amygdala and insula. While the activations in the right amygdala and anterior insula appeared stronger in the IAPS task, there was less variance in the OASIS task, which consisted solely of negative emotional scenes, resulting in more statistically significant activations. 

A meta-analysis of n-back tasks with neutral stimuli found activity in the bilateral middle frontal gyrus and left anterior insula (among other regions [35]), pointing to the activation in the right anterior insula in our data being attributable to the emotional nature of the stimuli at use. The activation elicited by EMOBACK in our data was in line with previous research from our group that found activation in the bilateral dlPFC and anterior insula as well as deactivation in a region in the rostral anterior cingulate cortex [28]. While we observed some activation in the left dlPFC, it was much less pronounced and not statistically significant. Previous studies have also reported a predominant involvement of the right compared with the left dlPFC in coping with emotional distractors in working memory tasks [26,36]. The exploratory independent sample *t*-tests between the tasks revealed that the EMOBACK task elicited significantly greater right dlPFC and bilateral anterior insula activity than any other of the studied tasks. This finding was plausible given that EMOBACK was the only task of those studied here that had a working memory component on top of the emotional stimulation and also the one with the shortest stimulus duration likely requiring greater attention. We did not find amygdala activation in response to the EMOBACK task. This was in line with previous results showing high cognitive effort in emotion-cognition-interference tasks reducing amygdala activation in response to emotional stimuli [36]. EMOBACK also used written words as stimulus material, which have consistently been shown to be associated with a lower probability of amygdala activation compared with emotional pictures [37] possibly due to a greater stimulus complexity of the latter [38]. The pregenual ACC is part of the default mode network, which is characterized by deactivation during goal-directed tasks but is activated in autobiographical and self-referential thought and social cognition [19,39]. However, the pgACC is also implied in the cognitive regulation of affect [20]. The deactivation found here might thus represent an interaction of both reduced activity due to a cognitive process and involvement in the regulation of activity in emotion-reactive brain regions. A previous study from our group concordantly found the pgACC to deactivate less in an emotional compared with a neutral condition of EMOBACK [28]. 

Our result regarding the FACES task eliciting amygdala activation distinctively was in line with meta-analytic findings on tasks using emotional faces [6,7] where several brain regions apart from the amygdala (e.g., fusiform gyrus) were significantly associated with viewing facial expressions of emotion but none of the other ROIs studied here. A robust engagement of the bilateral amygdala has been found in response to facial stimuli regardless of valence [7]. However, the amygdala is routinely implicated in orienting responses to behaviorally relevant stimuli, suggesting it is especially sensitive to the emotional content in facial expressions [12].

The two tasks studied here that used pictures of naturalistic emotional scenes elicited activations in the anterior insula and amygdala. The tasks using naturalistic scene stimuli were found to elicit a wider set of neural activations than the FACES task, which was in line with a meta-analytic comparison of these two types of tasks [6]. While this meta-analysis did not find an association of scenic emotional stimuli with activation in the anterior insula, a later meta-analysis did establish a robust association of anterior insula activation and emotional stimulation [17]. Although in direct comparison the IAPS task provoked a significantly greater activation in the right amygdala and right anterior insula, the OASIS task elicited more reliable (and significant) bilateral activation in both the amygdala and anterior insula. This might be due to the differences between the tasks: while the OASIS task showed only negative stimulus material to participants, the IAPS task used both positive and negative scenes. The representation of positive and negative emotions in the brain seems to largely overlap [40]; however, there have been reports of negative emotions eliciting stronger activation in the amygdala [41] and the insula [42] compared with positive emotions, which might explain the different activation patterns between the two tasks. It further seems plausible that showing aversive content exclusively, as in the OASIS task, might more likely induce a negative affective state compared with alternating between pictures of a positive and negative valence as in the IAPS task, hence triggering a more consistent neuronal response. As we did not collect ratings of subjective emotional experience during the tasks, we can only speculate about this relation. 

Our results from the direct comparison of the FACES and the OASIS tasks appear at odds with the only other study that compared passive viewing tasks of emotional facial expressions and scenes [43]. In this study, the anterior insula showed greater activity in response to emotional face stimuli compared with naturalistic scenes. The stimuli selection (a wider range of emotions including positive ones) and contrasts used (emotion-fixation), however, were different from the tasks studied here. It remains inconclusive whether the activation profiles we reported from the tasks studied here generalized with similar paradigms or were rather specific to the exact task. 

Our results demonstrated that the different paradigms elicited different activation profiles and could be used to address different aspects of emotion processing. The pattern of activation elicited by the EMOBACK task suggested that it was well suited for the study of emotion-cognition interactions in the anterior insula, pgACC and (right) dlPFC, as might be of interest, for example, in the study of depression and its treatment [44,45]. Investigators primarily interested in amygdala activations and its potential change in response to interventions could deduce from our results to employ the FACES task whereas the OASIS task showed a robust activation in the amygdala as well as the anterior insula, allowing for a broader study of brain regions involved in emotion processing. Our results were less conclusive about recommendations concerning the IAPS task. It might have the greatest face validity to assess emotion processing among the tasks studied here as it presented naturalistic scenes of both a positive and negative valence. However, in our sample, there was substantial variability in the neural response during the IAPS task performance and a statistically significant change in activation was found only in the right amygdala. 

Currently, a large variety of fMRI paradigms are in use for the study of emotion processing, limiting comparability between studies and impeding concise meta-analyses [9]. Therefore, it can be difficult to extract from the literature which paradigm is best suited for a specific research question especially as it has become clear that analytic choices can heavily impact the results of fMRI studies [46,47]. To allow for a comparison between fMRI tasks, it is crucial that the data are studied using the same analysis pipeline as we did here. We thus hope that the present results of activation elicited by different emotional fMRI paradigms in relevant pre-defined ROIs might provide guidance for planning studies on emotion processing. 

Ultimately, the field of affective and clinical neuroscience would profit from standardized task protocols for the study of certain brain regions or mental processes as it would grant an optimized comparability of results and meta-analytic synthesis. Recent studies have shown that the fMRI paradigms currently in use lack the reliability that would be needed for the use of fMRI as a biomarker in pathology and intervention research [48,49]. One potential remedy that has been discussed is increasing the amount of individual data collected (i.e., longer scan time) [50], which could be achieved relatively easily by collecting several runs of the tasks in question. 

The present study has limitations that need to be considered when interpreting the results. Although the region of interest approach does increase power compared with whole-brain analyses, the size of the available samples was quite small, especially considering that the effects reported for the emotion perception tasks were at best of moderate size (0.5 < Cohen’s d < 0.8 [47]). Our study thus had limited power to find ‘true’ effects and there was an increased likelihood of statistically significant results representing false positives. We did, however, apply Bonferroni correction to account for multiple testing, limiting the risk of false positive results. Nevertheless, it was a limitation of the present study that the acquisition site was not included in the statistical modelling of the data. The data analyzed for this study stemmed from different samples adding in-between subject variance and were collected on different MRI scanners (although with largely similar sequences). Therefore, we cannot rule out systematic variability in the data stemming from the acquisition set-up [51]. Studies investigating multi-site reliability of task-based fMRI found that a possible effect of the site on the data is likely small [52,53]. Nevertheless, it was a limitation of the present study that the acquisition site was not included in the statistical modelling of the data. We used a standard SPM12-based pre-processing pipeline relying on defaults. Although this approach is very common, there are now superior alternatives available, in particular fMRI prep, a robust pre-processing pipeline that combines optimal processing steps from different analytical software packages [54]. Our results might be weakened by the suboptimal pre-processing. The mean age of the samples studied differed notably and although a recent meta-analysis [1] did not find an effect of age on neural activations during emotion processing, such effects have been suggested [55,56] and might have influenced the differences between the activation pattern that we observed here. As all participants in this study were young to middle-aged, a possible impact of age differences on the data was likely small. Lastly, the samples we analyzed for this study were confined to males. While they have been scrutinized [57], there have been reports of gender differences in neural processes in emotion processing (e.g., [58]). Although the latest meta-analysis on the matter did not find a consistent difference in neural activation patterns in emotion processing tasks between men and women [1], previous meta-analyses found functional lateralization differences based on gender [41,59] such that a potentially limited generalizability of our findings should be considered. 

## 5. Conclusions

The present study found that four common emotional fMRI paradigms elicited different profiles of neural activation. The results suggested that the FACES task was most useful for the selective study of the amygdala whereas the OASIS task robustly activated the left anterior insula and bilateral amygdala. The EMOBACK task evoked activation in the right dlPFC and bilateral anterior insula and deactivation of the pgACC. These results are valuable to inform the planning of future studies and the eventual development of functional MRI biomarkers. 

## Data Availability

The data presented in this study are available on request from the corresponding author. The data are not publicly available due to privacy concerns with MRI data sharing.

## Supplementary Materials

The following are available online at https://www.mdpi.com/article/10.3390/brainsci11050525/s1, Figure S1: Example Stimuli, Table S1: Demographic variables and scanning sites for the three samples, Table S2 Task characteristics of the four emotion tasks, Table S3: MRI sequence parameters at the different sites, Table S4: Region of interest analyses for the four tasks.

## References

[1] García-García I., Kube J., Gaebler M., Horstmann A., Villringer A., Neumann J. (2016). Neural Processing of Negative Emotional Stimuli and the Influence of Age, Sex and Task-Related Characteristics. Neurosci. Biobehav. Rev..

[2] Wu M., Kujawa A., Lu L.H., Fitzgerald D.A., Klumpp H., Fitzgerald K.D., Monk C.S., Phan K.L. (2016). Age-Related Changes in Amygdala-Frontal Connectivity during Emotional Face Processing from Childhood into Young Adulthood. Hum. Brain Mapp..

[3] McTeague L.M., Rosenberg B., Lopez J.M., Carreon D., Huemer J., Jiang Y., Chick C., Eickhoff S., Etkin A. (2020). Identification of Common Neural Circuit Disruptions in Emotional Processing Across Psychiatric Disorders. Am. J. Psychiatry.

[4] Outhred T., Das P., Felmingham K., Bryant R., Nathan P., Malhi G., Kemp A. (2014). Impact of Acute Administration of Escitalopram on the Processing of Emotional and Neutral Images: A Randomized Crossover FMRI Study of Healthy Women. J. Psychiatry Neurosci. JPN.

[5] Enneking V., Dzvonyar F., Dück K., Dohm K., Grotegerd D., Förster K., Meinert S., Lemke H., Klug M., Waltemate L. (2020). Brain Functional Effects of Electroconvulsive Therapy during Emotional Processing in Major Depressive Disorder. Brain Stimulat..

[6] Sabatinelli D., Fortune E.E., Li Q., Siddiqui A., Krafft C., Oliver W.T., Beck S., Jeffries J. (2011). Emotional Perception: Meta-Analyses of Face and Natural Scene Processing. Neuroimage.

[7] Müller V.I., Höhner Y., Eickhoff S.B. (2018). Influence of Task Instructions and Stimuli on the Neural Network of Face Processing: An ALE Meta-Analysis. Cortex.

[8] Reisch L.M., Wegrzyn M., Kissler J., Woermann F.G., Bien C.G. (2020). Negative Content Enhances Stimulus-Specific Cerebral Activity during Free Viewing of Pictures, Faces, and Words. Hum. Brain Mapp..

[9] Riedel M.C., Yanes J.A., Ray K.L., Eickhoff S.B., Fox P.T., Sutherland M.T., Laird A.R. (2018). Dissociable Meta-analytic Brain Networks Contribute to Coordinated Emotional Processing. Hum. Brain Mapp..

[10] Villalta-Gil V., Hinton K.E., Landman B.A., Yvernault B.C., Perkins S.F., Katsantonis A.S., Sellani C.L., Lahey B.B., Zald D.H. (2017). Convergent Individual Differences in Visual Cortices, but Not the Amygdala across Standard Amygdalar FMRI Probe Tasks. Neuroimage.

[11] Lindquist K.A., Wager T.D., Kober H., Bliss-Moreau E., Barrett L.F. (2012). The Brain Basis of Emotion: A Meta-Analytic Review. Behav. Brain Sci..

[12] Whalen P., Davis F.C., Oler J.A., Kim H., Kim M.J., Neta M. (2009). Human amygdala responses to facial expressions of emotion. The Human Amygdala.

[13] Stuhrmann A., Suslow T., Dannlowski U. (2011). Facial Emotion Processing in Major Depression: A Systematic Review of Neuroimaging Findings. Biol. Mood Anxiety Disord..

[14] Gentili C., Cristea I.A., Angstadt M., Klumpp H., Tozzi L., Phan K.L., Pietrini P. (2016). Beyond Emotions: A Meta-Analysis of Neural Response within Face Processing System in Social Anxiety. Exp. Biol. Med..

[15] Craig A.D. (2002). How Do You Feel? Interoception: The Sense of the Physiological Condition of the Body. Nat. Rev. Neurosci..

[16] Zaki J., Davis J.I., Ochsner K.N. (2012). Overlapping Activity in Anterior Insula during Interoception and Emotional Experience. NeuroImage.

[17] Duerden E.G., Arsalidou M., Lee M., Taylor M.J. (2013). Lateralization of Affective Processing in the Insula. NeuroImage.

[18] Seeley W.W., Menon V., Schatzberg A.F., Keller J., Glover G.H., Kenna H., Reiss A.L., Greicius M.D. (2007). Dissociable Intrinsic Connectivity Networks for Salience Processing and Executive Control. J. Neurosci. Off. J. Soc. Neurosci..

[19] Raichle M.E. (2015). The Brain’s Default Mode Network. Annu. Rev. Neurosci..

[20] Palomero-Gallagher N., Hoffstaedter F., Mohlberg H., Eickhoff S.B., Amunts K., Zilles K. (2019). Human Pregenual Anterior Cingulate Cortex: Structural, Functional, and Connectional Heterogeneity. Cereb. Cortex.

[21] Vogt B.A. (2014). Submodalities of Emotion in the Context of Cingulate Subregions. Cortex J. Devoted Study Nerv. Syst. Behav..

[22] Phan K.L., Wager T., Taylor S.F., Liberzon I. (2002). Functional Neuroanatomy of Emotion: A Meta-Analysis of Emotion Activation Studies in PET and FMRI. NeuroImage.

[23] Tang W., Jbabdi S., Zhu Z., Cottaar M., Grisot G., Lehman J.F., Yendiki A., Haber S.N. (2019). A Connectional Hub in the Rostral Anterior Cingulate Cortex Links Areas of Emotion and Cognitive Control. eLife.

[24] Ochsner K.N., Silvers J.A., Buhle J.T. (2012). Functional Imaging Studies of Emotion Regulation: A Synthetic Review and Evolving Model of the Cognitive Control of Emotion. Ann. N. Y. Acad. Sci..

[25] Schweizer S., Satpute A.B., Atzil S., Field A.P., Hitchcock C., Black M., Barrett L.F., Dalgleish T. (2019). The Impact of Affective Information on Working Memory: A Pair of Meta-Analytic Reviews of Behavioral and Neuroimaging Evidence. Psychol. Bull..

[26] Van Dillen L.F., Heslenfeld D.J., Koole S.L. (2009). Tuning down the Emotional Brain: An FMRI Study of the Effects of Cognitive Load on the Processing of Affective Images. NeuroImage.

[27] Kohn N., Eickhoff S.B., Scheller M., Laird A.R., Fox P.T., Habel U. (2014). Neural Network of Cognitive Emotion Regulation—An ALE Meta-Analysis and MACM Analysis. Neuroimage.

[28] Grimm S., Weigand A., Kazzer P., Jacobs A.M., Bajbouj M. (2012). Neural Mechanisms Underlying the Integration of Emotion and Working Memory. Neuroimage.

[29] Võ M.L.H., Conrad M., Kuchinke L., Urton K., Hofmann M.J., Jacobs A.M. (2009). The Berlin Affective Word List Reloaded (BAWL-R). Behav. Res. Methods.

[30] Olszanowski M., Pochwatko G., Kuklinski K., Scibor-Rylski M., Lewinski P., Ohme R.K. (2015). Warsaw Set of Emotional Facial Expression Pictures: A Validation Study of Facial Display Photographs. Front. Psychol..

[31] Lang P.J., Bradley M.M., Cuthbert B.N. (1997). International Affective Picture System (IAPS): Technical Manual and Affective Ratings. NIMH Cent. Study Emot. Atten..

[32] Kurdi B., Lozano S., Banaji M.R. (2017). Introducing the Open Affective Standardized Image Set (OASIS). Behav. Res. Methods.

[33] Price C.J., Veltman D.J., Ashburner J., Josephs O., Friston K.J. (1999). The Critical Relationship between the Timing of Stimulus Presentation and Data Acquisition in Blocked Designs with FMRI. Neuroimage.

[34] Friston K.J., Holmes A.P., Price C.J., Büchel C., Worsley K.J. (1999). Multisubject FMRI Studies and Conjunction Analyses. Neuroimage.

[35] Wang H., He W., Wu J., Zhang J., Jin Z., Li L. (2019). A Coordinate-Based Meta-Analysis of the n-Back Working Memory Paradigm Using Activation Likelihood Estimation. Brain Cogn..

[36] Erk S., Kleczar A., Walter H. (2007). Valence-Specific Regulation Effects in a Working Memory Task with Emotional Context. Neuroimage.

[37] Costafreda S.G., Brammer M.J., David A.S., Fu C.H.Y. (2008). Predictors of Amygdala Activation during the Processing of Emotional Stimuli: A Meta-Analysis of 385 PET and FMRI Studies. Brain Res. Rev..

[38] Schlochtermeier L.H., Kuchinke L., Pehrs C., Urton K., Kappelhoff H., Jacobs A.M. (2013). Emotional Picture and Word Processing: An FMRI Study on Effects of Stimulus Complexity. PLoS ONE.

[39] Spreng R.N. (2012). The Fallacy of a “Task-Negative” Network. Front. Psychol..

[40] Lindquist K.A., Satpute A.B., Wager T.D., Weber J., Barrett L.F. (2016). The Brain Basis of Positive and Negative Affect: Evidence from a Meta-Analysis of the Human Neuroimaging Literature. Cereb. Cortex.

[41] Wager T.D., Phan K.L., Liberzon I., Taylor S.F. (2003). Valence, Gender, and Lateralization of Functional Brain Anatomy in Emotion: A Meta-Analysis of Findings from Neuroimaging. Neuroimage.

[42] Aldhafeeri F.M., Mackenzie I., Kay T., Alghamdi J., Sluming V. (2012). Regional Brain Responses to Pleasant and Unpleasant IAPS Pictures: Different Networks. Neurosci. Lett..

[43] Britton J.C., Taylor S.F., Sudheimer K.D., Liberzon I. (2006). Facial Expressions and Complex IAPS Pictures: Common and Differential Networks. Neuroimage.

[44] Korgaonkar M.S., Grieve S.M., Etkin A., Koslow S.H., Williams L.M. (2013). Using Standardized FMRI Protocols to Identify Patterns of Prefrontal Circuit Dysregulation That Are Common and Specific to Cognitive and Emotional Tasks in Major Depressive Disorder: First Wave Results from the ISPOT-D Study. Neuropsychopharmacology.

[45] Phillips M.L., Chase H.W., Sheline Y.I., Etkin A., Almeida J.R., Deckersbach T., Trivedi M.H. (2015). Identifying Predictors, Moderators, and Mediators of Antidepressant Response in Major Depressive Disorder: Neuroimaging Approaches. Am. J. Psychiatry.

[46] Botvinik-Nezer R., Holzmeister F., Camerer C.F., Dreber A., Huber J., Johannesson M., Kirchler M., Iwanir R., Mumford J.A., Adcock R.A. (2020). Variability in the Analysis of a Single Neuroimaging Dataset by Many Teams. Nature.

[47] Poldrack R.A., Baker C.I., Durnez J., Gorgolewski K.J., Matthews P.M., Munafò M.R., Nichols T.E., Poline J.B., Vul E., Yarkoni T. (2017). Scanning the Horizon: Towards Transparent and Reproducible Neuroimaging Research. Nat. Rev. Neurosci..

[48] Elliott M.L., Knodt A.R., Ireland D., Morris M.L., Poulton R., Ramrakha S., Sison M.L., Moffitt T.E., Caspi A., Hariri A.R. (2020). What Is the Test-Retest Reliability of Common Task-Functional MRI Measures? New Empirical Evidence and a Meta-Analysis. Psychol. Sci..

[49] Frohner J.H., Smolka M.N., Kroemer N.B., Teckentrup V. (2019). Addressing the Reliability Fallacy in FMRI: Similar Group Effects May Arise from Unreliable Individual Effects. Neuroimage.

[50] Nee D.E. (2019). FMRI Replicability Depends upon Sufficient Individual-Level Data. Commun. Biol..

[51] Jovicich J., Czanner S., Han X., Salat D., van der Kouwe A., Quinn B., Pacheco J., Albert M., Killiany R., Blacker D. (2009). MRI-Derived Measurements of Human Subcortical, Ventricular and Intracranial Brain Volumes: Reliability Effects of Scan Sessions, Acquisition Sequences, Data Analyses, Scanner Upgrade, Scanner Vendors and Field Strengths. Neuroimage.

[52] Gee D.G., McEwen S.C., Forsyth J.K., Haut K.M., Bearden C.E., Addington J., Goodyear B., Cadenhead K.S., Mirzakhanian H., Cornblatt B.A. (2015). Reliability of an FMRI Paradigm for Emotional Processing in a Multisite Longitudinal Study. Hum. Brain Mapp..

[53] Forsyth J.K., McEwen S.C., Gee D.G., Bearden C.E., Addington J., Goodyear B., Cadenhead K.S., Mirzakhanian H., Cornblatt B.A., Olvet D.M. (2014). Reliability of Functional Magnetic Resonance Imaging Activation during Working Memory in a Multi-Site Study: Analysis from the North American Prodrome Longitudinal Study. Neuroimage.

[54] Esteban O., Markiewicz C.J., Blair R.W., Moodie C.A., Isik A.I., Erramuzpe A., Kent J.D., Goncalves M., DuPre E., Snyder M. (2019). FMRIPrep: A Robust Preprocessing Pipeline for Functional MRI. Nat. Methods.

[55] Cacioppo J.T., Berntson G.G., Bechara A., Tranel D., Hawkley L.C. (2011). Could an aging brain contribute to subjective well-being? The value added by a social neuroscience perspective. Social Neuroscience: Toward Understanding the Underpinnings of the Social Mind.

[56] MacCormack J.K., Stein A.G., Kang J., Giovanello K.S., Satpute A.B., Lindquist K.A. (2020). Affect in the Aging Brain: A Neuroimaging Meta-Analysis of Older Vs. Younger Adult Affective Experience and Perception. Affect. Sci..

[57] Bluhm R. (2013). New Research, Old Problems: Methodological and Ethical Issues in FMRI Research Examining Sex/Gender Differences in Emotion Processing. Neuroethics.

[58] Wrase J., Klein S., Gruesser S.M., Hermann D., Flor H., Mann K., Braus D.F., Heinz A. (2003). Gender Differences in the Processing of Standardized Emotional Visual Stimuli in Humans: A Functional Magnetic Resonance Imaging Study. Neurosci. Lett..

[59] Stevens J.S., Hamann S. (2012). Sex Differences in Brain Activation to Emotional Stimuli: A Meta-Analysis of Neuroimaging Studies. Neuropsychologia.

