# Supplementary material for: Comparison of Four fMRI Paradigms Probing Emotion Processing
Source: Brain Sci. 2021 Apr 21;11(5):525. doi: 10.3390/brainsci11050525 (PMC8142995; doi:10.3390/brainsci11050525)
Supplement: Supplementary file 1 [file brainsci-11-00525-s001.zip › brainsci-1161826-supplementary.pdf]

Supplementary Material

Supplementary Table S1 Demographic variables and scanning site for the three samples

	EMOBACK	FACES & OASIS*	IAPS
N	15	15	15
Gender	All male	All male	All male
Age	29.3 (±2.9)	25.8 (±5.3)	35.5 (±10.8)
Scanning site (n)	CCNB (15)	BCAN (15)	CCNB/UZH (5/10)

*FACES and OASIS task were assessed in the same sample; CCNB = Center for Cognitive Neuroscience Berlin, BCAN= Berlin Center for Advanced Neuroimaging; UZH = University of Zurich

Supplementary Table S2 Task characteristics of the four emotion tasks

	IAPS	EMOBACK	OASIS	Faces
Task design	block design	block design	block design	block design
Conditions	Positive/negative	Positive/negative/ neutral	negative/scrambled	negative/scrambled
Contrast of interest	Emotional - break	Emotional – break	Negative – scrambled	Negative – scrambled
Stimulus modality	Picture	Word	Picture	Picture
Stimuli database	IAPS	BAWL	OASIS	WSEFEP
Num. of conditions	2	3	2	2
Total num. of stimuli	80	225	84	144
Num. of blocks	16	15	28	24
Blocks per condition	8	5	14	12
Stimuli per block	5	15	3	6
Block duration	20s	21s	18s	18s
Stimuli per condition	40	75	42	72
Stimulus duration	4s	500ms	6s	3s
Inter-stimulus interval	none	1500ms	none	none
Break duration	20 s	10-14s	20-21s	20-21s
Attention check	Question for 8s after each block	n-back task	Indicate whether person is in the picture/ frame colour	Indicate gender/frame colour
break	fixation cross	fixation cross	fixation cross	fixation cross
Overall duration	13min	12min	15min	15min
Volumes collected	384	331	430	370

Supplementary Table S3 MRI sequence parameters at the different sites.

	BCAN	UZH	CCNB
Manufacturer, model name, field strength	Siemens MAGNETOM Prisma 3T	Philips Achieva TX 3T	Siemens MAGNETOM TrioTim 3T
Receiver coil	12-channel head coil	8-channel head coil	12-channel head coil
**Functional imaging**			
Imaging type	EPI	EPI	EPI
TE	30ms	35ms	30ms
TR	2000ms	2000ms	2000ms
Flip angle	80°	82°	70°
Number of slices placed along the anterior–posterior commissure plane	36	32	37
Slice thickness	3mm	4mm	3mm
FOV	192mm	220mm	192mm
Voxel size	3x3x3 mm	2.75 x 2.75 x 4 mm	3x3x3mm
Parallel imaging method	GRAPPA	SENSE	GRAPPA
Slice order	Interleaved	Ascending	Interleaved
Fat suppression	Fat saturation	Fat saturation	Fat saturation
**Anatomical imaging**			
Imaging type	3D	3D	3D
TR/TE	3.03	4.6	2.52
TR	2300ms	9.3ms	1900ms
Flip angle	9°	8°	9°
Number of slices	192	160	176
FOV	256x256mm	240x240mm	256x256mm
Voxel size	1x1x1mm	1x1x1mm	1x1x1mm

Supplementary Table S4 Region of interest analyses for the four tasks. Mean parameter estimates standard deviation, parameters for two-sided Student t-test (t, df, p) and confidence intervals.

A. EMOBACK

	leftAInsula	leftAM	leftDLPFC	pgACC	rightAInsula	rightAM	rightDLPFC
Mean β	1.48495	-0.24744	0.47400	-1.04277	1.32879	-0.28052	1.93647
SD	0.49629	0.50044	0.68464	0.55353	0.57498	0.55892	0.79502
t	11.58833	-1.91496	2,68142	-7,29613	8,95062	-1,94384	9,43357
df	14	14	14	14	14	14	14
p	0.00001	0.07616	0.01790	0.00001	0.00001	0.07229	0.00001
95% CI	[1.21012, 1.75979]	[-0.52458, 0.0297]	[0.09486, 0.85314]	[-1.3493, -0.73623]	[1.01038. 1.64720]	[-0.59004, 0.029]	[1.49620, 2.37674]

B. FACES

	leftAInsula	leftAM	leftDLPFC	pgACC	rightAInsula	rightAM	rightDLPFC
Mean β	0.09323	0.46985	-0.03039	0.03257	-0.00293	0.46597	-0.09403
SD	0.16553	0.31467	0.16785	0.22641	0.16341	0.33638	0.16507
t	2.18137	5.78297	-0.70129	0.55709	-0.06952	5.36501	-2.20631
df	14	14	14	14	14	14	14
p	0.04670	0.00005	0.49461	0.58627	0.94556	0.00010	0.04457
95% CI	[0.00156, 0.18490]	[0.29559, 0.64410]	[-0.12335, 0.06256]	[-0.09282, 0.15795]	[-0.09343, 0.08756]	[0.27969, 0.65225]	[-0.18544, -0.00262]

C. OASIS

	leftAInsula	leftAM	leftDLPFC	pgACC	rightAInsula	rightAM	rightDLPFC
Mean β	0.29931	0.43119	0.05705	0.08167	0.16307	0.42283	0.03093
SD	0.29854	0.27543	0.20688	0.31179	0.20431	0.32088	0.27998
t	3.88297	6.06322	1.06807	1.01444	3.09125	5.10350	0.42790
df	14	14	14	14	14	14	14
p	0.00166	0.00003	0.30356	0.32758	0.00797	0.00016	0.67523
95% CI	[0.13399, 0.46464]	[0.27866, 0.58372]	[-0.05752, 0.17162]	[-0.091, 0.25433]	[0.04993, 0.27622]	[0.24513, 0.60052]	[-0.12411, 0.18598]

D. IAPS

	leftAInsula	leftAM	leftDLPFC	pgACC	rightAInsula	rightAM	rightDLPFC
Mean β	0.40364	0.64603	0.20267	-0.33096	0.36585	0.89803	0.24453
SD	0.68117	0.82985	0.64881	0.62744	0.46560	0.80244	0.69763
t	2.29500	3.01507	1.20983	-2.04289	3.04322	4.33437	1.35752
df	14	14	14	14	14	14	14
p	0.03771	0.00927	0.24638	0.06036	0.00877	0.00069	0.19610

95% CI	[0.02642, 0.78086]	[0.18647, 1.10558]	[-0.15663, 0.56197]	[-0.67843, 0.01651]	[0.10801, 0.62369]	[0.45366, 1.34241]	[-0.14181, 0.63086]

S Table 5 Exploratory independent sample t-test (two-sided) parameters for contrasts between mean parameter estimates for tasks collected in independent samples.

A. pgACC

Contrast	EMOBACK / IAPS	EMOBACK / FACES	EMOBACK / OASIS	IAPS / FACES	IAPS / OASIS
df	28	28	28	28	28
t	-1.944	-7.286	-6.779	-2.111	-2.281
p	0.062	<.001	<.001	.044	.030
Cohen's d	.734	2.753	2.562	.798	.862

B. right dlPFC

Contrast	EMOBACK / IAPS	EMOBACK / FACES	EMOBACK / OASIS	IAPS / FACES	IAPS / OASIS
df	28	28	28	28	28
t	4.669	9.579	8.107	1.829	1.100
p	<.001	<.001	<.001	.078	.280
Cohen's d	1.765	3.6216	3.064	.691	.41

C. left dlPFC

Contrast	EMOBACK / IAPS	EMOBACK / FACES	EMOBACK / OASIS	IAPS / FACES	IAPS / OASIS
df	28	28	28	28	28
t	.556	2.598	1.898	.135	.828
p	.583	.015	.068	.188	.415
Cohen's d	.210	.982	.718	.509	.313

D. right anterior insula

Contrast	EMOBACK / IAPS	EMOBACK / FACES	EMOBACK / OASIS	IAPS / FACES	IAPS / OASIS
df	28	28	28	28	28
t	3.412	8.106	6.385	2.894	1.544
p	.002	<.001	<.001	.007	.134
Cohen's d3	1.292	3.064	2.413	1.094	.583

E. left anterior insula

Contrast	EMOBACK / IAPS	EMOBACK / FACES	EMOBACK / OASIS	IAPS / FACES	IAPS / OASIS
df	28	28	28	28	28
t	3.037	9.111	5.933	1.715	.543
p	.005	<.001	<.001	.097	.591
Cohen’s d	1.148	3.443	2.243	.648	.205

F. right amygdala

Contrast	EMOBACK / IAPS	EMOBACK / FACES	EMOBACK / OASIS	IAPS / FACES	IAPS / OASIS
df	28	28	28	28	28
t	-4.912	-5.424	-5.193	1.923	2.130
p	<.001	<.001	<.001	.065	.042
Cohen’s d	1.857	2.050	1.963	.727	.805

G. left amygdala

Contrast	EMOBACK / IAPS	EMOBACK / FACES	EMOBACK / OASIS	IAPS / FACES	IAPS / OASIS
df	28	28	28	28	28
t	-3.709	-6.264	-6.360	.769	.952
p	<.001	<.001	<.001	.448	.349
Cohen’s d	1.402	2.368	2.404	.291	.360

s

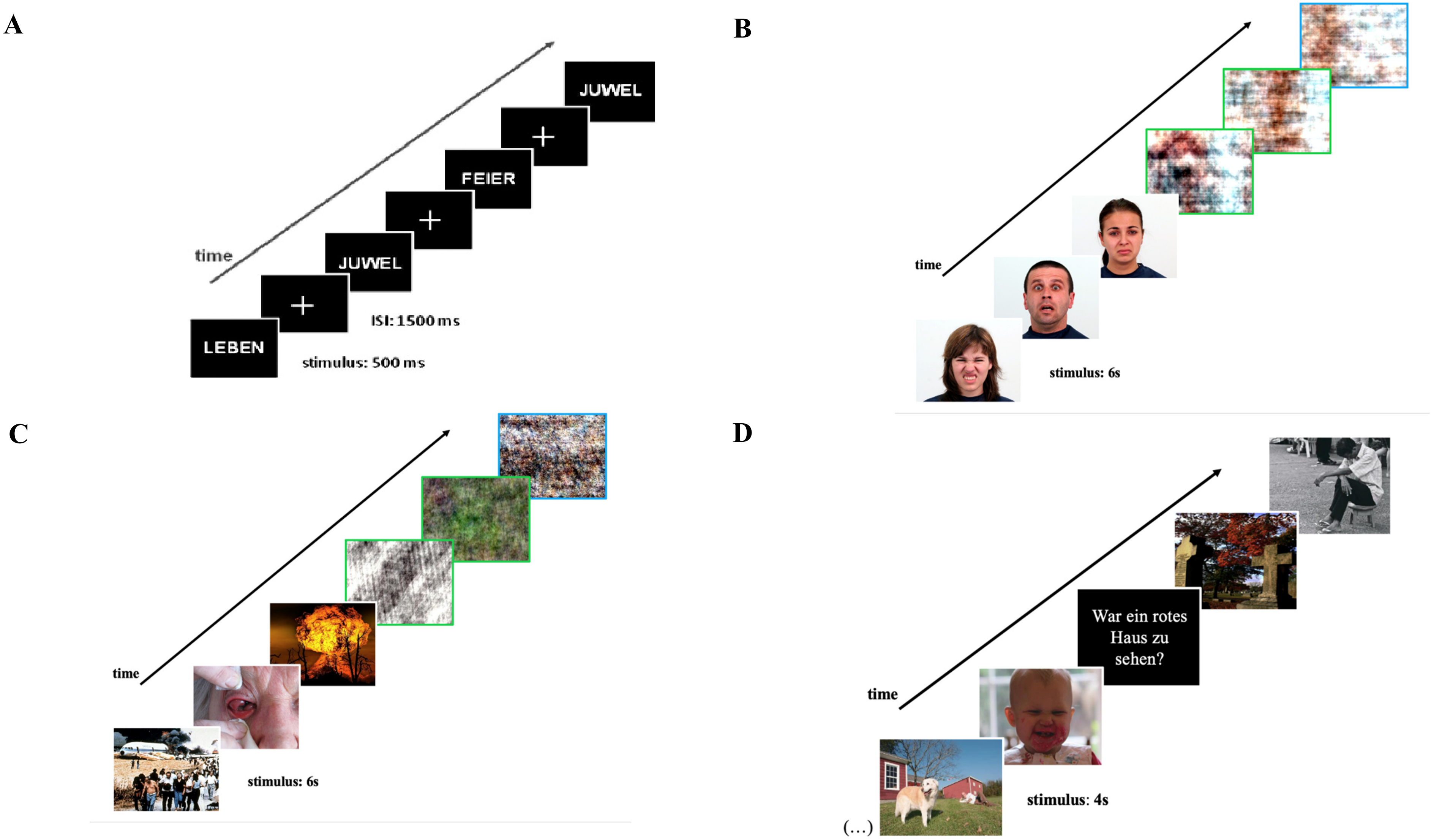

Figure S1. Example stimuli for (**A**) EMOBACK (**B**) FACES (**C**) OASIS and (**D**) IAPS task.
